# *CHEK2* variants in susceptibility to breast cancer and evidence of retention of the wild type allele in tumours

**DOI:** 10.1038/sj.bjc.6600637

**Published:** 2002-11-26

**Authors:** N Sodha, S Bullock, R Taylor, G Mitchell, B Guertl-Lackner, R D Williams, S Bevan, K Bishop, S McGuire, R S Houlston, R A Eeles

**Affiliations:** Royal Marsden NHS Trust, Downs Road, Sutton, Surrey SM2 5PT, UK; Section of Cancer Genetics, Institute of Cancer Research, Sutton, Surrey SM2 5NG, UK; Medical Genetics Unit, St George's Hospital, London, SW17 ORE, UK; Section of Molecular Carcinogenesis, Institute of Cancer Research, Sutton, Surrey SM2 5NG, UK; Section of Paediatric Oncology, Institute of Cancer Research, Sutton, Surrey SM2 5NG, UK

**Keywords:** breast cancer, *CHEK2*, mutation

## Abstract

We have recently shown that the CHEK2*1100delC mutation acts as a low penetrance breast cancer susceptibility allele. To investigate if other *CHEK2* variants confer an increased risk of breast cancer, we have screened an affected individual with breast cancer from 68 breast cancer families. Five of these individuals were found to harbour germline variants in *CHEK2*. Three carried the 1100delC variant (4%). One of these three individuals also carried the missense variant, Arg180His. In the other two individuals, missense variants, Arg117Gly and Arg137Gln, were identified. These two missense variants reside within the Forkhead-associated domain of *CHEK2,* which is important for the function of the expressed protein. None of these missense variants were present in 300 healthy controls. Microdissected tumours with a germline mutation showed loss of the mutant allele suggesting a mechanism for tumorigenesis other than a loss of the wild type allele. This study provides further evidence that sequence variation in *CHEK2* is associated with an increased risk of breast cancer, and implies that tumorigenesis in association with *CHEK2* mutations does not involve loss of the wild type allele.

*British Journal of Cancer* (2002) **87**, 1445–1448. doi:10.1038/sj.bjc.6600637 www.bjcancer.com

© 2002 Cancer Research UK

## 

About 10% of breast cancer has a genetic predisposition, however, germline mutations in *BRCA1,* *BRCA2*, *TP53, PTEN* and *LKB1* only account for between 20–25% of the familial clustering (Easton, 1999). A recent model of breast cancer susceptibility suggests a polygenic basis for the wide variation in familial risk whereby the individually small effects of many genes combine multiplicatively (Antoniou *et al*, 2001). We have recently found evidence for this model with the observation that the 1100delC variant in *CHEK2* which is carried by ∼1% of the population confers a 1.7-fold increased risk of breast cancer (Meijers-Heijboer *et al*, 2002).

*CHEK2* functions downstream of ATM (ataxia telangiectasia-mutated protein) in response to DNA damage (Chaturvedi *et al*, 1999) to phosphorylate TP53 (Chehab *et al*, 2000) and *BRCA1* (Lee *et al*, 2000), therefore regulating the tumour suppressor functions of these proteins. The protein contains functionally important 60 amino acid FHA domain (residues 115–175) and a kinase domain (residues 226–486) (Matsuoka *et al*, 1998).

To investigate if variants of *CHEK2* other than 1100delC confer an increased risk of breast cancer, we have screened a series of 68 familial breast cancer cases, which had been screened in the Regional Genetics Service and found to be negative for mutations in *BRCA1* and *BRCA2*.

## MATERIALS AND METHODS

### Patients

EDTA-venous blood samples were collected from 68 familial breast cancer cases attending Genetics Clinics within the Royal Marsden NHS Trust and the South Thames Regional Genetics Unit. All families had at least two individuals affected with breast cancer. The breast cancer was verified by histological reports. Samples were obtained with informed consent and local ethical approval in accordance with the tenets of the Helsinki Declaration. DNA was extracted from EDTA-blood samples using a standard sucrose lysis method.

## METHODS

### Mutational analysis of *BRCA1* and *BRCA2*

Mutational analysis of *BRCA1* and *BRCA2* was undertaken using a combination of the protein truncation test (PTT) (Hogervorst *et al*, 1995), and conformation sensitive gel electrophoresis (CSGE) (Ganguly *et al*, 1993). Exons 10 and 11 of *BRCA2* and exon 11 of *BRCA1* were screened by PTT. Exon 9 of *BRCA2* and exons 2 and 20 of *BRCA1* were screened by CSGE.

### Mutational analysis of *CHEK2*

The search for germline mutations in *CHEK2* was performed using CSGE as described previously (Sodha *et al*, 2002). Samples that showed variant migration bands were sequenced by direct sequencing using the ABI BigDye cycle sequencing kit with dye-labelled terminators. These labelled products were then run on an ABI 310 sequencer (Applied Biosystems).

### Loss of heterozygosity (LOH) studies

Allelic imbalance in tumours from individuals with germline mutations in *CHEK2* was assessed using the microsatellite marker, D22S275, which maps to intron 4 of *CHEK2*. The relevant exons were also amplified by PCR from the tumour DNA and sequenced. DNA was obtained from paraffin-embedded tissue by dewaxing with xylene, digesting with 10 mM Tris-HCl (pH 7.5), 1 mM EDTA, 15% (w v^−1^) SDS and 500 μg ml^−1^ proteinase K for 4 h at 56°C, followed by phenol-chloroform extraction and sodium acetate, ethanol precipitation.

### Statistical analysis

The 95% confidence interval (95% CI) of the estimate of the frequency of *CHEK2* mutations in breast cancer cases was estimated from the binomial distribution. All statistical manipulations were undertaken using the statistical software programme STATA (Version 6.0, Stata Corporation, College Station, Texas, TX 77840, USA; http://www.stata.com).

## RESULTS

Table 1

**Table 1:**
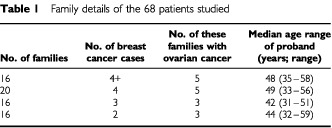
Family details of the 68 patients studied

shows the family details of the 68 breast cancer cases studied. All had a strong family history of breast cancer or breast-ovarian cancer. The median age at diagnosis of all cases was 47 years.

Seventeen of the 68 patients were fully screened for mutations in *BRCA1* and *BRCA2*. The others were screened for mutations in exons 9–11 of *BRCA2* and exons 2, 11 and 20 of *BRCA1*. No pathogenic mutations were detected in any of the 68 patients. This analysis is estimated to detect approximately 75% of the reported *BRCA1* mutations and 50% of the *BRCA2* gene. (BIC database: www.nhgri.nih.gov/Intramural_research/Lab_transfer/Bic/).

**Figure 1:**
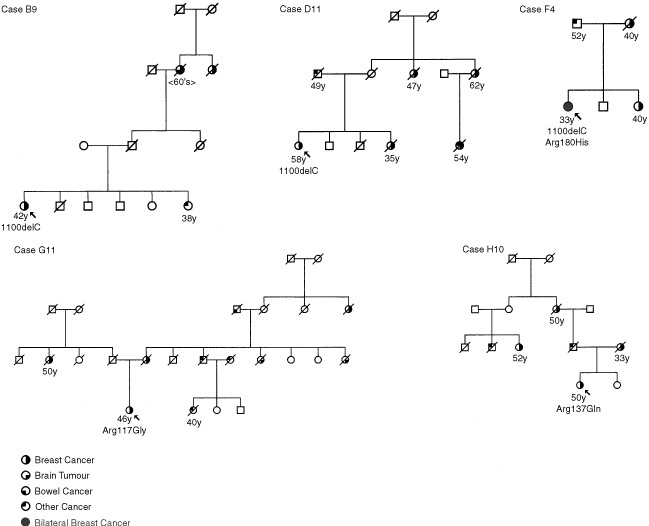
The pedigrees of the breast cancer cases with germline mutations in *CHEK2* (individuals harbouring the mutation are arrowed). The trees have been altered to preserve anonymity, but not alter the meaning of the report.

The full coding sequence of *CHEK2* was screened for mutations in all 68 patients. Six sequence variants were identified. Figure 1

shows the family histories of the individuals carrying the variants. Three of the cases, B9, F4 and D11, carried the 1100delC variant. One of these cases, F4, also carried the missense variant, Arg180His. The other two individuals, G11 and H10, carried sequence changes in exon 2 – Arg117Gly and Arg137Gln.

To establish whether the *CHEK2* missense variants identified represent polymorphisms, 300 healthy controls were screened for these sequence changes. None of the controls were found to harbour these changes. Unfortunately, germline DNA was not available from other members in any of the three families to examine whether either variant was carried by any other family member affected with breast cancer.

**Figure 2:**
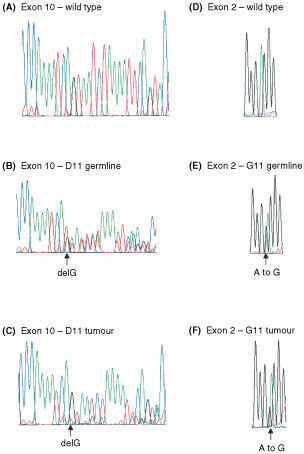
Partial sequences of exon 10 and exon 2 of *CHEK2*. (**A**) wild type reverse sequence of exon 10 (**B**) germline reverse sequence of exon 10 from case D11 (**C**) reverse sequence of exon 10 from tumour DNA from case D11 (**D**) wild type forward sequence of exon 2 (**E**) germline forward sequence of exon 2 from case G11 (**F**) forward sequence of exon 2 from tumour DNA from case G11. The wild type allele is retained and there is a low level signal of the mutant allele in both the sequences of tumour DNA in (**C**) and (**F**) (arrowed).

Paraffin embedded tissue was available from the two patients with the 1100delC variant (B9 and D11) and the patient with the Arg117Gly variant (G11). Analysis of tumour DNA from B9 showed no evidence of allelic imbalance by sequencing. Sequencing of relevant exons from tumour DNA from D11 and G11, however, showed that the mutant allele was lost and the wild type allele retained (Figure 2

).

Figure 3

**Figure 3:**
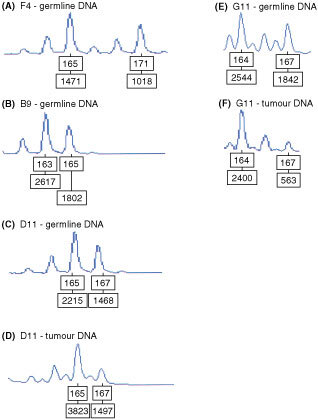
Genescans of the microsatellite marker D22S275. (**A**–**C**) Germline DNA of individuals with the 1100delC variant (**D**) tumour DNA of case D11 (**E**) germline DNA of case G11 who harbours the variant Arg117Gly and (**F**) tumour DNA of G11. There is a loss of heterozygosity in tumour DNA from both the cases.

shows D22S275 genotypes of patients F4, B9 and paired normal -tumour DNA samples from patients D11 and G11. We have previously shown that the 165bp allele of D22S275 is associated with the 1100delC variant (Meijers-Heijboer *et al*, 2002) and in this study all three patients who harbour this sequence change (B9, F4, D11) possessed this allele. Figure 3 shows evidence of LOH in the tumour DNA from patients G11 and D11. However, there was retention of the 165bp allele in patient D11, who carried the del1100C variant.

## DISCUSSION

In our study, three of the 68 breast cancer patients (4%; 95% CI: 1–12%), carried the 1100delC variant which we have previously reported to act as a low-penetrance breast cancer susceptibility allele (Meijers-Heijboer *et al*, 2002). One of these individuals also carried the missense variant, Arg180His. Two other missense variants were also detected in the patients analysed, Arg117Gly and Arg137Gln. Neither of these was detected in a large series of controls implying that they may have a role in the aetiology of breast cancer. On the assumption that they have pathogenic potential, variations in *CHEK2* may account for around 7% (95%; CI: 2–16%) of familial breast cancer cases.

Two previously reported studies have analysed the whole of *CHEK2* in familial breast cancer – Allinen *et al* (2001) studied 79 hereditary breast cancer cases and Sullivan *et al* (2002) examined 45 familial breast cancer cases. Neither found evidence to support a role of sequence variation in *CHEK2* in familial breast cancer. However, both studies failed to show that del1100C confers an increased risk of breast cancer.

One of the missense sequence changes we identified involved Arg117 of *CHEK2*. Arg117 resides within the FHA domain of *CHEK2* and is conserved through evolution (Matsuoka *et al*, 1998). Tumour DNA from the individual with this variant was available to investigate whether loss of heterozygosity is a mechanism by which this variant might confer susceptibility. Allelic imbalance was detected, but the wild type allele was retained and the mutant allele lost. It is possible that the wild type allele has acquired a more lethal somatic mutation and therefore the mutant allele has been preferentially lost or that this variant causes cancer by a mechanism other than by loss of the wild type allele.

Ahn *et al* (2002) have recently shown that some mutations in the FHA domain negatively affect activation of *CHEK2*. In response to ionising radiation, ATM phosphorylates Thr-68 of *CHEK2* (Ahn *et al*, 2000). *CHEK2* is then autophosphorylated on Thr-383 and Thr-387 in a phospho-threonine 68 (Thr(P)-68)-dependent manner (Lee and Chung, 2001). Ahn *et al* (2002) have shown that phosphorylation of Thr-68 promotes oligomerisation of *CHEK2* by serving as a specific ligand for the FHA domain of another *CHEK2* molecule. Only catalytically inactive *CHEK2* forms oligomers. Ahn *et al* (2002) have postulated that activation of *CHEK2* occurs through oligomerisation of *CHEK2* via binding of the Thr-68 phosphorylated region of one *CHEK2* to the FHA domain of another. Oligomerisation of *CHEK2* therefore increases the efficiency of transautophosphorylation resulting in the release of active *CHEK2* monomers that proceed to enforce checkpoint control. These authors have also shown that Arg117Ala mutation negatively affects autophosphorylation by significantly reducing the ability of *CHEK2* to bind to thr (P)-68 molecule. Since the mutation we identified, Arg117Gly, is also a non-conservative substitution it is highly likely to behave in the same way.

Tumour DNA from two patients with the 1100delC variant was also available to assess allelic imbalance. In one of the 1100delC cases no LOH was observed. In the other case, D11, as in the case with the Arg117Gly variant, sequencing of exon 10 showed that the wild type allele was retained. However, in this individual there was retention of the microsatellite 165 bp allele that is associated with 1100delC. It is possible that a recombination event may have taken place between the 1100del variant and the D22S275allele.

The CHEK2*1100delC mutation results in truncation of the protein at codon 381. Hence the functional segment of the protein encompassing amino acids T383 and T387 which are responsible for autophosphorylation will be lost. It is conceivable that if oligomerisation takes place between one normal peptide and one truncated peptide, transautophosphorylation may be not be possible and the normal *CHEK2* molecules may still remain bound to the truncated molecules reducing the concentration of the active monomers to respond to DNA damage. Our present investigation suggests that a few rare variants in *CHEK2* may confer an increased susceptibility to breast cancer. On the assumption that all the variants we have identified have pathogenic potential then variation in *CHEK2* might account for 7% (95% CI indicate up to 16%) of familial breast cancer. The way in which mutations in *CHEK2* cause breast cancer is likely to be through mechanisms other than the loss of heterozygosity that is observed with other classical tumour suppresser genes.
